# Increased levels and activation of the IL-17 receptor in microglia contribute to enhanced neuroinflammation in cerebellum of hyperammonemic rats

**DOI:** 10.1186/s40659-024-00504-2

**Published:** 2024-04-27

**Authors:** Yaiza M. Arenas, Adrià López-Gramaje, Carmina Montoliu, Marta Llansola, Vicente Felipo

**Affiliations:** 1 Laboratory of Neurobiology, Centro de Investigación Príncipe Felipe, Eduardo Primo-Yufera 3, 46012 Valencia, Spain; 2 Departamento de Patología, Facultad de Medicina, Universidad Valencia, Valencia, Spain; 3 INCLIVA Instituto de Investigación Sanitaria, Valencia, Spain

**Keywords:** Cerebellum, Hyperammonemia, IL-17, Neuroinflammation, Microglia, Hepatic encephalopathy

## Abstract

**Background:**

Patients with liver cirrhosis may show minimal hepatic encephalopathy (MHE) with mild cognitive impairment and motor incoordination. Rats with chronic hyperammonemia reproduce these alterations. Motor incoordination in hyperammonemic rats is due to increased GABAergic neurotransmission in cerebellum, induced by neuroinflammation, which enhances TNFα-TNFR1-S1PR2-CCL2-BDNF-TrkB pathway activation. The initial events by which hyperammonemia triggers activation of this pathway remain unclear. MHE in cirrhotic patients is triggered by a shift in inflammation with increased IL-17. The aims of this work were: (1) assess if hyperammonemia increases IL-17 content and membrane expression of its receptor in cerebellum of hyperammonemic rats; (2) identify the cell types in which IL-17 receptor is expressed and IL-17 increases in hyperammonemia; (3) assess if blocking IL-17 signaling with anti-IL-17 ex-vivo reverses activation of glia and of the TNFα-TNFR1-S1PR2-CCL2-BDNF-TrkB pathway.

**Results:**

IL-17 levels and membrane expression of the IL-17 receptor are increased in cerebellum of rats with hyperammonemia and MHE, leading to increased activation of IL-17 receptor in microglia, which triggers activation of STAT3 and NF-kB, increasing IL-17 and TNFα levels, respectively. TNFα released from microglia activates TNFR1 in Purkinje neurons, leading to activation of NF-kB and increased IL-17 and TNFα also in these cells. Enhanced TNFR1 activation also enhances activation of the TNFR1-S1PR2-CCL2-BDNF-TrkB pathway which mediates microglia and astrocytes activation.

**Conclusions:**

All these steps are triggered by enhanced activation of IL-17 receptor in microglia and are prevented by ex-vivo treatment with anti-IL-17. IL-17 and IL-17 receptor in microglia would be therapeutic targets to treat neurological impairment in patients with MHE.

## Introduction

Cirrhotic patients may show hepatic encephalopathy (HE) which may lead to coma and death. More than 40% of cirrhotic patients show minimal hepatic encephalopathy (MHE) with psychomotor slowing, mild cognitive impairment and motor incoordination which strongly reduces their quality of life and life span. Hyperammonemia is a main contributor to the cognitive and motor impairment in patients with MHE [1–3]. One of the earliest alterations in MHE is motor incoordination [4]. Motor incoordination is a consequence of enhanced GABAergic neurotransmission in cerebellum, both in patients [5] and in animal models [6]. Increased GABAergic neurotransmission is due to neuroinflammation, with enhanced activation of the TNFα-TNFR1-NF-kB-glutaminase-GAT3 pathway, which results in increased extracellular GABA [7–9] and of the TNFα-TNFR1-S1PR2-CCL2-BDNF-TrkB pathway which results in increased content of the GABA synthesizing enzymes GAD65 and GAD67 and of GABA and increased membrane expression of GABA_A_ receptors and of the chloride co-transporter KCC2 [10, 11]. However, the initial events by which chronic hyperammonemia triggers the activation of these pathways in cerebellum remain unclear.

It has been shown that MHE in cirrhotic patients is triggered by a shift in peripheral inflammation to a profile similar to that observed in autoimmune diseases, with increased activation of Th17 lymphocytes and of IL-17 in plasma [12, 13]. These patients also show neuroinflammation in cerebellum [14]. IL-17 receptor is expressed in microglia and, at much lower levels, in astrocytes [15].

Chen et al. [16] showed that, in a model of chronic migraine, peripheral IL-17 may cross the blood–brain barrier and reach the brain, where it activates microglia and induces neuroinflammation. In retinal vascular diseases there is an increase of IL-17 and of its receptor which result in enhanced activation of microglia which increases pro-inflammatory factors and contributes to the main pathological changes in retinal vascular diseases [17]. Chen et al. [16] propose that blockade of IL-17 could prevent vascular damages.

A key role of IL-17 in triggering neuroinflammation and pathological alterations has been reported in different neurodegenerative diseases [18, 19].

The studies mentioned in the introduction suggest that increased IL-17 in cerebellum may play a role in the enhanced neuroinflammation, microglia activation and activation of TNFα-TNFR1-S1PR2-CCL2-BDNF-TrkB pathways in cerebellum of hyperammonemic rats. The aims of this work were: )1) assess if hyperammonemia increases the content of IL-17 and membrane expression of its receptor in cerebellum of hyperammonemic rats; (2) identify the cell types in which IL-17 receptor is expressed and IL-17 increases; (3) assess if blocking IL-17 signaling with anti-IL-17 ex vivo reverses activation of microglia and astrocytes and TNFα-TNFR1-S1PR2-CCL2-BDNF-TrkB pathways in cerebellum of hyperammonemic rats. The antibody used in the present work recognizes IL-17A, therefore when we refer to IL-17 along the article we are referring to IL-17A.

## Materials and methods

### Rats

Male Wistar rats (220–250 g) were made hyperammonemic by feeding them a diet containing ammonium acetate for 4–5 weeks as in Felipo et al. [20] modified to contain 25% instead of 20% ammonium acetate as in Taoro-Gonzalez et al. [21].The experiments were approved by the Comite Ético de Experimentación Animal (CEEA) of our center (2019–12) and by the Conselleria de Agricultura of Generalitat Valenciana (2019/VSC/PEA/0224), were performed in accordance with the guidelines of the Directive of the European Commission (2010/63/EU) for care and management of experimental animals, and comply with the ARRIVE guidelines for animal research. A total of 56 rats were used in this study, 28 controls and 28 hyperammonemic.

### Analysis of protein content and phosphorylation in cerebellar slices by Western blot

Control and hyperammonemic rats were sacrificed at 4 weeks of hyperammonemia. Cerebellar slices (400 μm-thick, transversal) were cut and 1 µg/ml of anti-IL-17A (ABCAM Ref: AB79056) was added to slices and incubated for 30 min. Samples were subjected to Western blot as in Felipo et al., [22] using antibodies against IL-17A (1:1000) and TrkB (1:500) from ABCAM, TNFα (1:1000) from R&D systems, CCL2 (1:1000) fron Proteintech, BDNF (1:1000) from Invitrogen and actin or GAPDH as a control for protein loading. Hyperammonemia increases IL-17 levels, as reported in the results section.

### Analysis of membrane expression of receptors and transporters

Membrane expression of proteins in cerebellar slices was analyzed by cross-linking with BS3 (Pierce cat# 21580, Rockford, IL) as described in Cabrera-Pastor et al. [23]. Slices were added to tubes containing ice-cold Krebs buffer with or without 2 mM bis(sulfosuccinimidyl)suberate (BS3) (Pierce, Rockford, IL, USA) and incubated for 30 min at 4 °C. Cross-linking was terminated by adding 100 mM glycine (10 min, 4 °C). The slices were homogenized by sonication for 20 s. Samples treated with or without BS3 were analysed by Western blot using antibodies against using antibodies against IL-17 Receptor, TrkB, TNFR1 (1:500) from ABCAM, S1PR2 (1:1000) from Proteintek, CCR2 (1:1000) from Novus, P2X4 (1:1000) from Invitrogen and KCC2 (1:1000) from Millipore. BS3 is a cross-linker that reacts with proteins in the membrane surface generating aggregates that do not enter the gel. So that the band in the cytosol is reduced compared to the sample without BS3. The surface expression of receptor subunits was calculated as the difference between the intensity of the bands without BS3 (total protein) and with BS3 (non-membrane protein) [23].

### Immunohistochemistry

After the incubation of cerebellar slices with anti-IL-17A, slices were fixed in 4% paraformaldehyde in 0.1 M phosphate buffer (pH 7.4) during 24 h at 4 °C. Paraffin-embedded slices were incubated with antibodies against Iba1 (Wako; 1:300), GFAP (SIGMA; 1:400), IL-17A (ABCAM; 1:200), IL-17RA (ABCAM; 1:100), TNFα (ABCAM; 1:200) overnight. Then, slides were incubated with Goat anti-mouse or anti-rabbit (biotinylated) secondary antibodies (Vector Laboratories) for 1 h and diaminobenzidine for 10 min. Sections were counterstained with Mayer’s hematoxylin (DAKO) for 5 min. Once the slides were dry, they were scanned using an Aperio Versa scanner (Leica Biosystems Nussloch GmbH, Germany). Scanned slides were analyzed using ImageScope64 software, which allows photos of areas of interest to be obtained at different magnifications. In all the Immunohistochemistry analyses we include a control without primary antibody where no signal is seen, indicating that the staining reflects true binding of the antibodies.

### Analysis of astrocytes and microglia activation

Analysis of Iba1 staining was performed in two regions of the cerebellum white matter and molecular layer and the analysis of GFAP was performed in the white matter of cerebellar slices using the Image J software as in Arenas et al. [24]. Cerebellar slices from four to six animals per group were used. Usually 4 rats per group is enough to obtain statistically significant results. However, in some cases 6 rats are necessary to reach statistical significance.

### Analysis of IL-17A and TNFα content in Purkinje neurons and granular layer

Analysis of staining of IL-17A or TNFα was performed in Purkinje neurons using the Image J software. Purkinje neurons were manually selected using freehand selection of ROI manager function and the mean intensity (M.I.) of staining for IL-17 was recorded. The results are expressed as Integrated Optical Density (IOD). In the case of the granular layer, the area stained by the antibody against each protein (IL-17A or TNFα) is manually marked with the Image Pro-Plus program and the mean optical density of the stained area is obtained. The analysis was performed on at least 10 40x-fields for each rat.

### Immunofluorescence analysis of the IL-17 receptor, pSTAT3, IL-17A and TNFα in astrocytes and microglia

Double immunofluorescences were performed to analyze IL-17RA (1:50, ABCAM), IL-17A (1:100, ABCAM) and TNFα (1:150, ABCAM) co-localization with astrocytes (using GFAP, 1:400, SIGMA) and microglia (using Iba1, 1:300, ABCAM). Double immunofluorescences were performed to analyze pSTAT3 (Tyr705) (1:100, Cell Signaling), co-localization with microglia (using Iba1, 1:300, ABCAM). Analysis of IL-17RA, IL-17A, TNFα or pSTAT3 (Tyr705) staining was performed in the white matter of the cerebellum using the Image J software. The number of cells expressing each protein in white matter of the cerebellum was manually counted using the Cell Counter plugin of ImageJ and the results are expressed as cells/mm^2^. The analysis for the region was performed on at least 10 40x-fields for each rat.

### Immunofluorescence analysis of subcellular localization of NF-κB p50 in Purkinje neurons, and of NF-κB p50 content in microglia, astrocytes and granular layer

Analysis of the p50 subunit of NF-κB was performed by immunofluorescence as in Arenas and Felipo, 2023 [24]. Sections from six different animals from each group were selected, washed in 0.1 M phosphate buffer, and blocked with normal serum from the same species as the secondary antibody before being incubated overnight with primary antibodies (NF-κB p50 (1:100), and Iba-1 (1:300); ABCAM; GFAP (1:400); Sigma-Aldrich) diluted in blocking buffer and fluorescent secondary antibodies (1:400; Invitrogen). The nuclei were counterstained with DAPI (Sigma-Aldrich), and the sections were coverslipped. The sections were observed under a confocal microscope (Leica TCS-SP2-AOBS) and imaged.

In the Purkinje neurons, the p50 subunit may be located in the nucleus or cytosol. The nuclear and cytoplasmic intensities of the p50 subunit were analyzed using ImageJ (1.48v). Nuclei was outlined on the blue (DAPI) channel using the ROI manager function, and the selection was applied on the green channel (p50) to measure fluorescence. The mean intensity (M.I.) for each nucleus was measured. For analysis of cytoplasmic NF-κB p50 subunit, the green channel was used; the cytosol of each cell was manually outlined using the freehand selection tool of ImageJ, and the mean intensity (M.I.) was recorded. The results are expressed as the nuclear/cytoplasmic ratios of the p50 subunit of NF-κB.

Double immunofluorescence of the microglia marker Iba-1 or the astrocytes marker GFAP and the p50 subunit of NF-κB were performed to analyze the expression of NF-κB p50 in these glial cell types.

### Statistical analysis

Results are expressed as mean ± standard error. All statistical analyses were performed using the software program GraphPad Prism v. 9.0. Normality was assessed using the D’Agostino and Pearson Omnibus test and the ShapiroWilk normality tests. Differences in variances of normally distributed data were assessed using Bartlett’s test. Data with the same variance across groups were analyzed by a parametric one-way analysis of variance (ANOVA) followed by Fisher’s LSD multiple comparisons test or two‐way ANOVA when appropriate. A confidence level of 95% was accepted as significant. The number of rats used for each parameter and the statistical procedure used in each case is indicated in the corresponding figure legend.

## Results

### Hyperammonemia increases the content of IL-17 and of the IL-17 receptor and membrane expression of the receptor in cerebellum

**Fig. 1:**
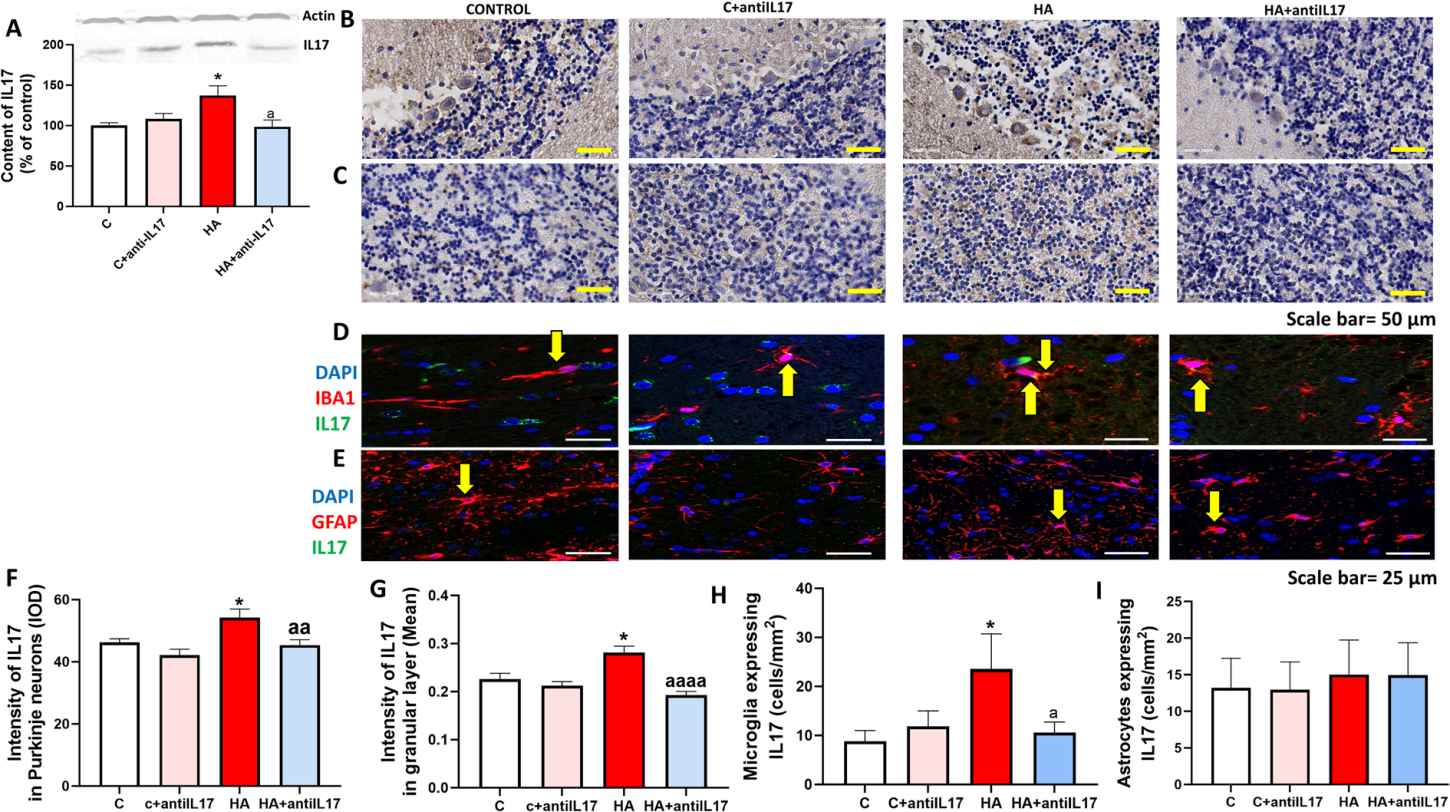
Hyperammonemia increases the content of IL-17 in Purkinje neurons, granular neurons and microglia but not in astrocytes. Ex vivo treatment with anti-IL-17 reversed the increase of IL-17 (**A**–**E**). The total content of IL-17 in whole cerebellum of control (**C**) and hyperammonemic (HA) rats was analyzed by Western blot (**A**). The effects of hyperammonemia and of blocking IL-17 with anti-IL-17 in Purkinje neurons (**B**, **F**) and granular neurons (**C**, **G**) was analyzed by immunohistochemistry with DAB staining using antibodies against IL-17 and was quantified as described in Methods (**F**, **G**). Representative images are shown in **B**, **C**. Double fluorescence staining was performed using anti-IL-17 and anti-IBA1 (**D**, **H**) or GFAP (**E**, **I**) as described in methods. Representative images of the double fluorescence staining are shown (**D**, **E**). Values are mean ± SEM of 9–25 rats per group in A and 4–6 rats per group in B–E. One-way ANOVA followed by Fisher’s LSD post-hoc test was performed to compare all groups. Values are the mean ± SEM. Values significantly different from control group are indicated by asterisk (*p < 0.05) and values significantly different from HA group are indicated by a (a = p < 0.05, aa = p < 0.01, aaaa = p < 0.0001). Yellow arrows indicates co-localization of the two proteins

We analyzed the content of IL-17 in whole cerebellum by Western blot. As shown in Fig. 1A, hyperammonemia increases (p < 0.001) IL-17 content in cerebellum.

We analyzed by immunohistochemistry the cell types in which IL-17 is increased in hyperammonemic rats. Chronic hyperammonemia increased IL-17 levels in Purkinje neurons (p < 0.01, Fig. 1B and F), in granular neurons (p < 0.05, Fig. 1C and G) and in microglia (p < 0.05, Fig. 1D and H), but not in astrocytes (Fig. 1E and I). Ex vivo treatment of cerebellar slices from hyperammonemic rats with anti-IL-17 completely reversed the increase of IL-17 content in Purkinje and granular neurons and in microglia (Fig. 1B-D, F–H).

**Fig. 2:**
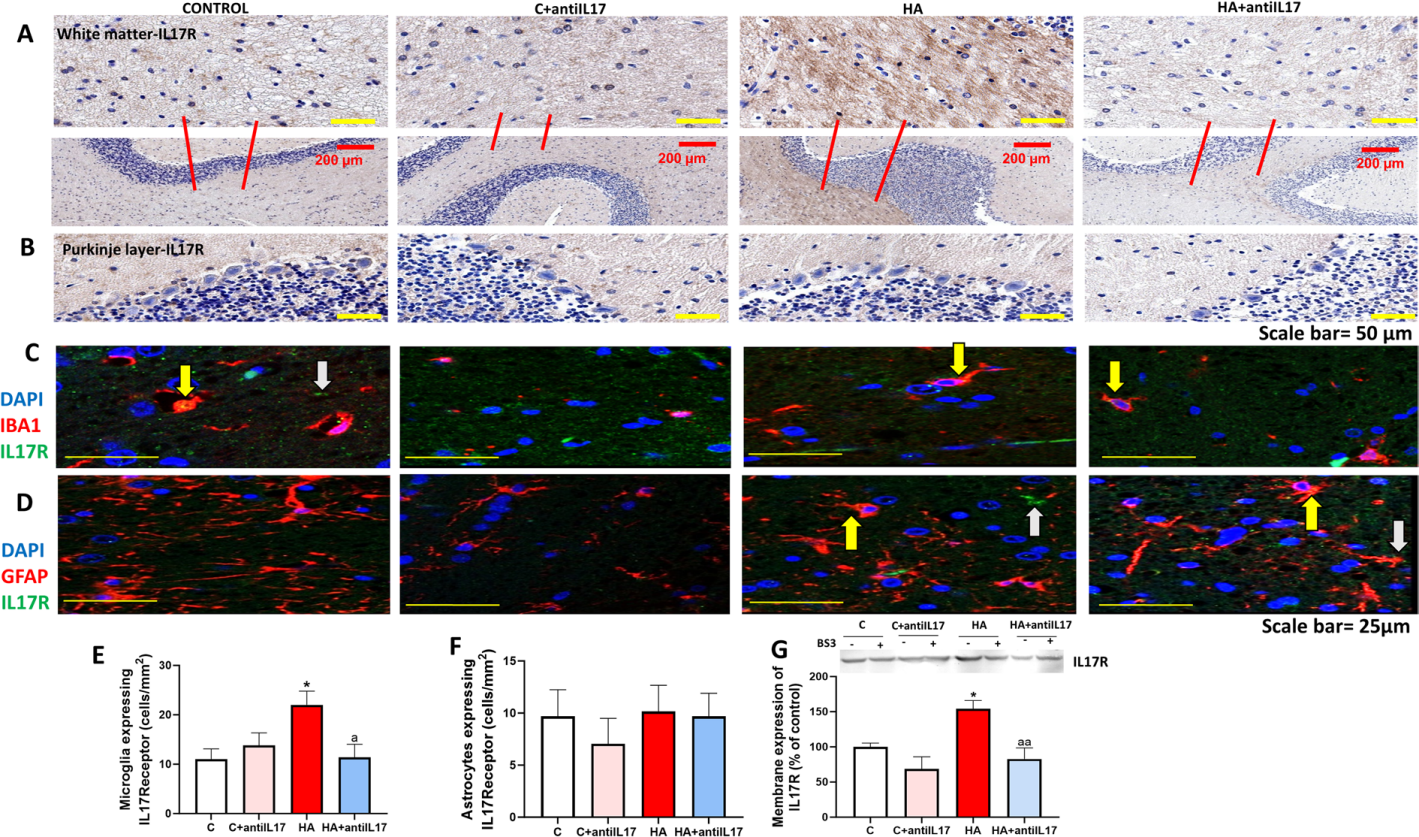
Hyperammonemia increases the content of IL-17 receptor in microglia and its membrane expression in cerebellum. Blocking IL-17 with anti-IL-17 reverses these effects. Membrane expression of the IL-17 receptor (G) was analyzed using BS3 cross-linker in slices from control (C) and hyperammonemic (HA) rats. The effects of hyperammonemia and of blocking IL-17 with anti-IL-17 in white matter (**A**) and Purkinje neurons (**B**) were analyzed by immunohistochemistry with DAB staining using antibodies against IL-17 receptor. Representative images are shown in A-B. The content of IL-17 receptor in Purkinje neurons and granular neurons was negligible. Double immunofluorescence staining was performed using anti-IL-17 receptor and anti-IBA1 (**C**, **E**) or GFAP (**D**, **F**). Representative images of the double fluorescence staining are shown (**C**, **D**). Values are mean ± SEM of 10–20 rats per group in G and 4–6 rats per group in **A**–**D**. One-way ANOVA followed by Fisher’s LSD post-hoc test was performed to compare all groups. Values are the mean ± SEM. Values significantly different from control group are indicated by asterisk (*p < 0.05) and values significantly different from HA group are indicated by a (a = p < 0.05, aa = p < 0.01). Yellow arrows indicate co-localization of the two proteins while white arrows indicate lack of co-localization. As the IL-17 receptor is expressed both in astrocytes and microglia, there are some instances of double immunofluorescence in which the markers (Iba1 or GFAP) and the IL-17 receptor do not co-localize effectively. We indicate this situation with white arrows. For example, in **C** there are some cells that are stained by anti-IL-17R but not by anti-Iba1. These cells may be for example astrocytes, which also express IL-17R. The same occurs in **D**, some cells (likely microglia) are stained by anti-IL-17R but not by anti-GFAP

The effects of IL-17 would be mediated by activation of its receptor, we therefore also analyzed the effects of hyperammonemia on the IL-17 receptor. We first analyzed in which cell types is the IL-17 receptor expressed and altered by hyperammonemia. The expression of the IL-17 receptor is high in white matter and very low in the Purkinje layer (Fig. 2A and B). The receptor is mainly expressed in glial cells of white matter (Fig. 2A). To assess the effects of hyperammonemia on the IL-17 receptor in microglia and astrocytes we performed double immunofluorescences of IL-17 receptor with Iba1 (marker of microglia) or GFAP, marker of astrocytes. Hyperammonemia increased the content of the IL-17 receptor in microglia (Fig. 2C and E), but not in astrocytes (Fig. 2D and F). The increase of the IL-17 receptor in microglia was reversed by ex vivo incubation with anti-IL-17 (Fig. 2C and E). In Figs. 2C and D we indicate with yellow arrows co-localization of the IL-17 receptor with microglia (Fig. 2C) or with astrocytes (Fig. 2D). As the IL-17 receptor is expressed both in astrocytes and microglia, there are some instances of double immunofluorescence in which the markers (Iba1 or GFAP) and the IL-17 receptor do not appear to co-localize effectively. We indicate this situation with white arrows. For example, in Fig. 2C there are some cells that are stained by anti-IL-17R but not by anti-Iba1. These cells may be for example astrocytes, which also express IL-17R. The same occurs in Fig. 2D, some cells (likely microglia) are stained by anti-IL-17R but not by anti-GFAP.

As only the receptor expressed in the membrane surface may be activated by extracellular IL-17, we analyzed membrane expression of the receptor by Western blot using the crosslinker BS3. Hyperammonemia also increased the membrane expression of the IL-17 receptor in cerebellum (Fig. 2H). These data support that IL-17 signaling is increased in cerebellum of hyperammonemic rats, especially in microglia, due to increased levels of IL-17, of the total amount of IL-17 receptor and of the membrane expression of the receptor.

### Blocking IL-17 with anti-IL-17 reduces activation of astrocytes and microglia in cerebellum of hyperammonemic rats

**Fig. 3:**
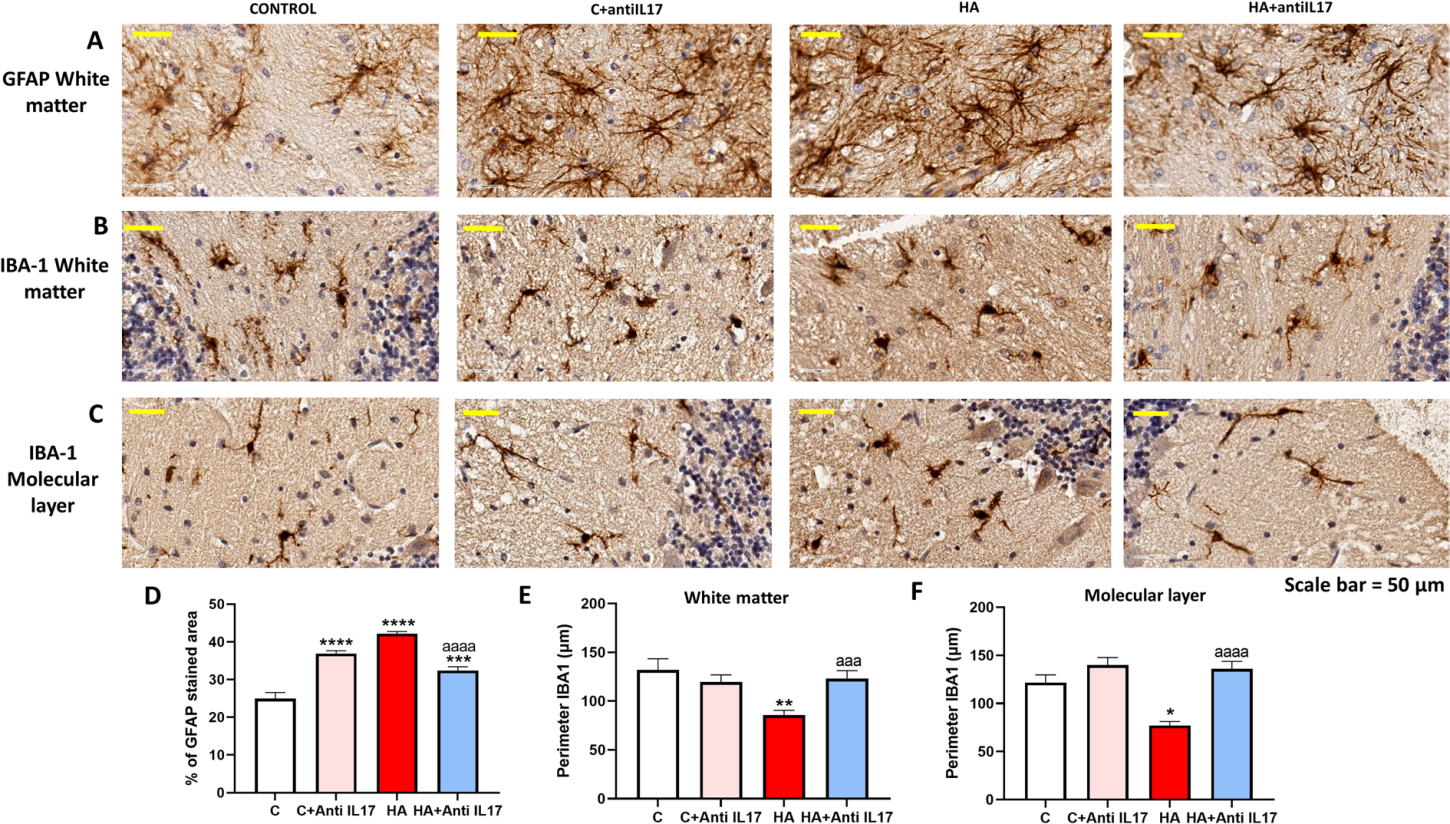
Ex vivo incubation with anti-IL-17 reduces astrocytes and microglia activation in cerebellar slices from hyperammonemic rats. Representative images of immunohistochemistry against GFAP (**A**), IBA1 in white matter (**B**) and IBA1 in molecular layer (**C**) in cerebellar slices. GFAP stained area expressed as percentage of total area (**D**), the perimeter of microglia (IBA1 stained cells) in white matter (**E**) and molecular layer (**F**) are expressed in μm (n = 4–6). One-way ANOVA followed by Fisher’s LSD post-hoc test was performed to compare all groups. Values are the mean ± SEM. Values significantly different from control group are indicated by asterisk (*p < 0.05, *p < 0.01, ****p < 0.0001) and values significantly different from HA group are indicated by a (aaa = p < 0.001, aaaa = p < 0.0001)

We then assessed if increased IL-17 signaling contributes to maintain neuroinflammation in cerebellum of hyperammonemic rats. Hyperammonemic rats show neuroinflammation with activation of astrocytes and microglia. Astrocytes are activated in the cerebellar white matter of hyperammonemic rats, as indicated by the increase in the area stained with GFAP (Fig. 3A). In hyperammonemic rats the GFAP-stained area increased to 42 ± 0.5% (p < 0.0001) (Fig. 1D) compared to 25 ± 2% in control rats, indicating astrocyte activation in hyperammonemia. Treatment with anti-IL-17 ex vivo reversed (p < 0.0001) the increase in GFAP-stained area, reducing it to 32 ± 1%, indicating that anti-IL-17 reverses astrocyte activation in hyperammonemic rats (Fig. 3A and D).

We also analyzed microglia activation by measuring its perimeter, which is reduced when microglial cells become activated. Microglia is activated in white matter and molecular layer of cerebellum of hyperammonemic rats. In the cerebellar white matter and molecular layer, the perimeter of microglia of hyperammonemic rats decreased (p < 0.001 and p < 0.01) to 86 ± 5 µm and 77 ± 4 µm compared to 132 ± 11 µm and 117 ± 7 µm in control rats. Treatment with anti-IL-17 ex vivo reversed microglia activation, increasing the perimeter to 123 ± 8 µm (p < 0.001) and 136 ± 8 µm (p < 0.0001), respectively (Fig. 3B, C, E, F).

### Blocking IL-17 with anti-IL-17 reverses enhanced activation of the TNFα-S1PR2-CCL2-BDNF-TrkB pathway in cerebellum of hyperammonemic rats

**Fig. 4:**
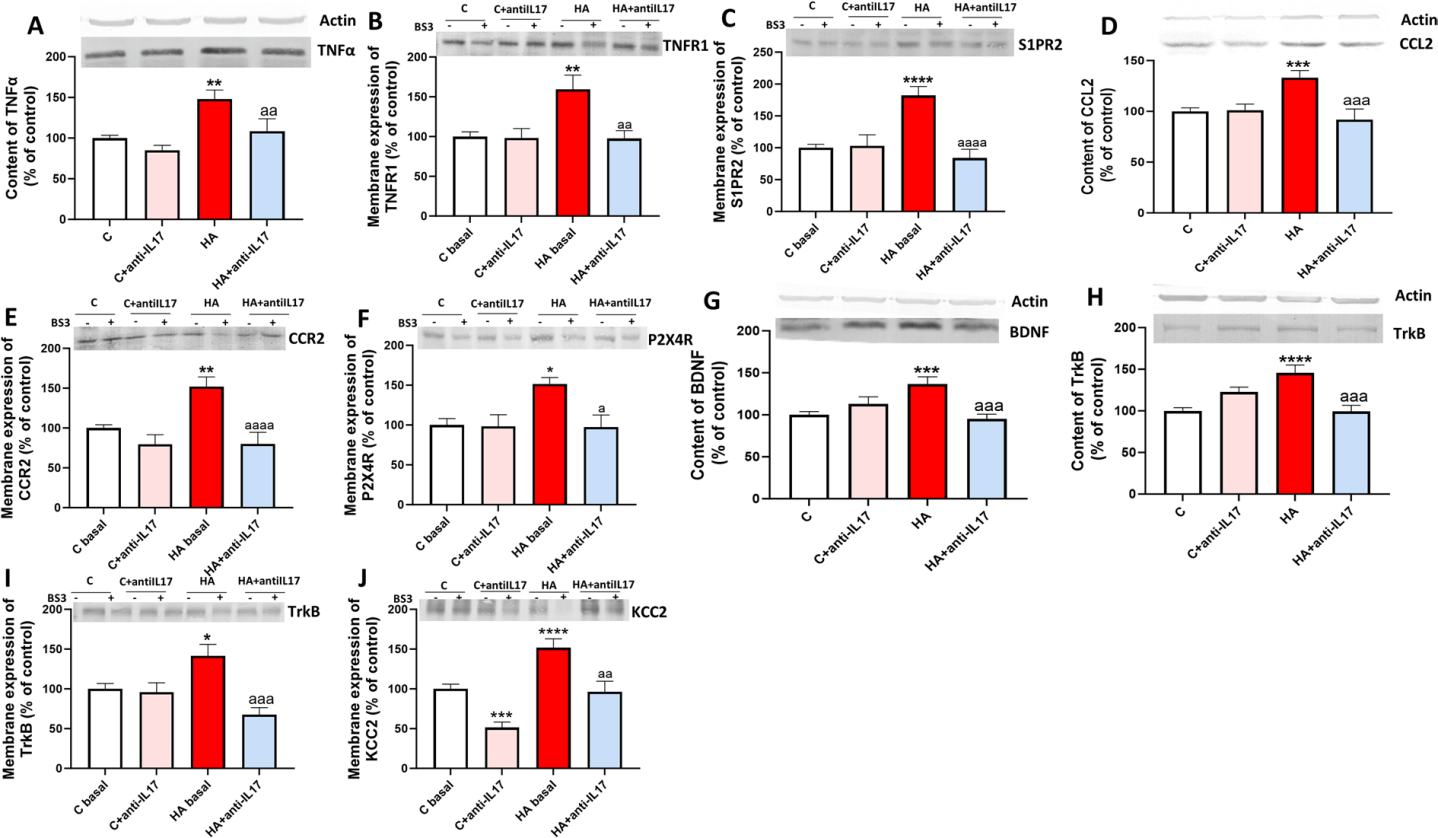
Ex vivo treatment with anti-IL-17 reverse the alterations in the TNFα-TNFR1-S1PR2-CCL2-BDNF-TrkB pathway in cerebellar slices from hyperammonemic rats. Protein content of (**A**) TNFα (n = 20–30), (**D**) CCL2 (n = 17–27), (**G**) BDNF (n = 18–28) and (**H**) TrKB (n = 22–26) in cerebellar slices, assessed by Western blot. Membrane expression of (**B**) TNFR1 (n = 17–35), (**C**) S1PR2 (n = 19–27), (**E**) CCR2 (n = 20–30), (**F**) P2X4R (n = 12–20), (**I**) TrKB (n = 12–25) and (**J**) KCC2 (n = 16–27) was analyzed using BS3 cross-linker. Representative images of the blots of each protein are shown. Values are expressed as percentage of controls and are the mean ± SEM. One-way ANOVA followed by Fisher’s LSD post-hoc test was performed to compare all groups. Values significantly different from controls are indicated by asterisk (*p < 0.05, **p < 0.01, ***p < 0.001, ****p < 0.0001) and values significantly different from HA group are indicated by a (a = p < 0.05, aa = p < 0.01, aaa = p < 0.001, aaaa = p < 0.0001)

We have recently shown that enhanced activation of the TNFα-S1PR2-CCL2-BDNF pathway contributes to sustained neuroinflammation and to enhancement of GABAergic neurotransmission in cerebellum of hyperammonemic rats [10, 11]. We assessed if blocking IL-17 ex vivo in cerebellar slices with anti-IL-17 reverses the activation of this pathway. Anti-IL-17 reverses the increase in TNFα (Fig. 4A), in the membrane expression of the TNFR1 (Fig. 4B), S1PR2 (Fig. 4C), in the content of CCL2 (Fig. 4D), in the membrane expression of its receptor CCR2 (Fig. 4E) and of P2X4 (Fig. 4F), in the content of BDNF (Fig. 4G) and in the content (Fig. 4H) and membrane expression (Fig. 4I) of its receptor TrkB and of the chloride co-transporter KCC2 (Fig. 4J).

### Hyperammonemia increases NF-κB and TNFα content in microglia, astrocytes and neurons. Anti-IL-17 reverses these increases

**Fig. 5:**
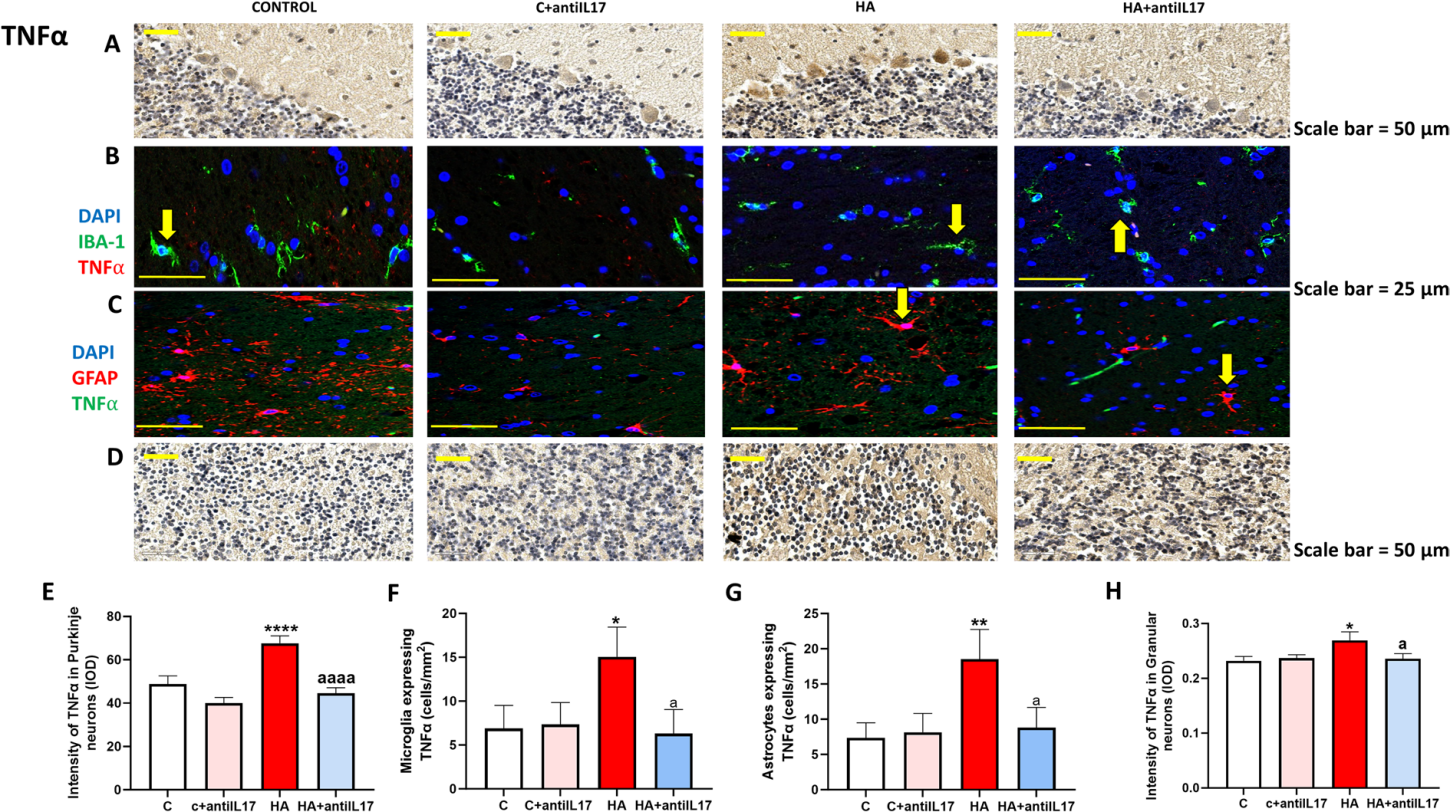
Hyperammonemia increases the content of TNFα in Purkinje neurons, granular neurons, microglia and astrocytes in cerebellum. Ex vivo treatment with anti-IL-17 reverses the increase of TNFα in Purkinje neurons (**A**, **E**) and granular neurons (**D**, **H**) as analyzed by immunohistochemistry with DAB staining using antibodies against TNFα. Double fluorescence staining was performed using anti-TNFα and anti-IBA1 (**B**, **F**) or GFAP (**C**, **G**). Representative images of the immunohistochemistry and double fluorescence staining are shown (**A**–**D**). Values are mean ± SEM of 4–6 rats per group. One-way ANOVA followed by Fisher’s LSD post-hoc test was performed to compare all groups. Values are the mean ± SEM. Values significantly different from control rats are indicated by asterisk (*p < 0.05, **p < 0.01, ****p < 0.0001) and values significantly different from HA group are indicated by a (a = p < 0.05, aaaa = p < 0.0001). Yellow arrows mean colocalization of the two proteins

The IL-17-induced increase in TNFα is key in triggering the activation of the TNFα-S1PR2-CCL2-BDNF pathway. To advance in the understanding of the cell types involved in triggering this pathway we analyzed in which cell types is the content of TNFα increased by hyperammonemia and reduced by anti-IL-17. TNFα content increased in Purkinje neurons (Fig. 5A), microglia (Fig. 5B) and astrocytes (Fig. 5C) and granular cells (Fig. 5D) of hyperammonemic rats. Anti-IL-17 treatment reversed the increase of TNFα in all these types of cells (Fig. 5E–H).

**Fig. 6:**
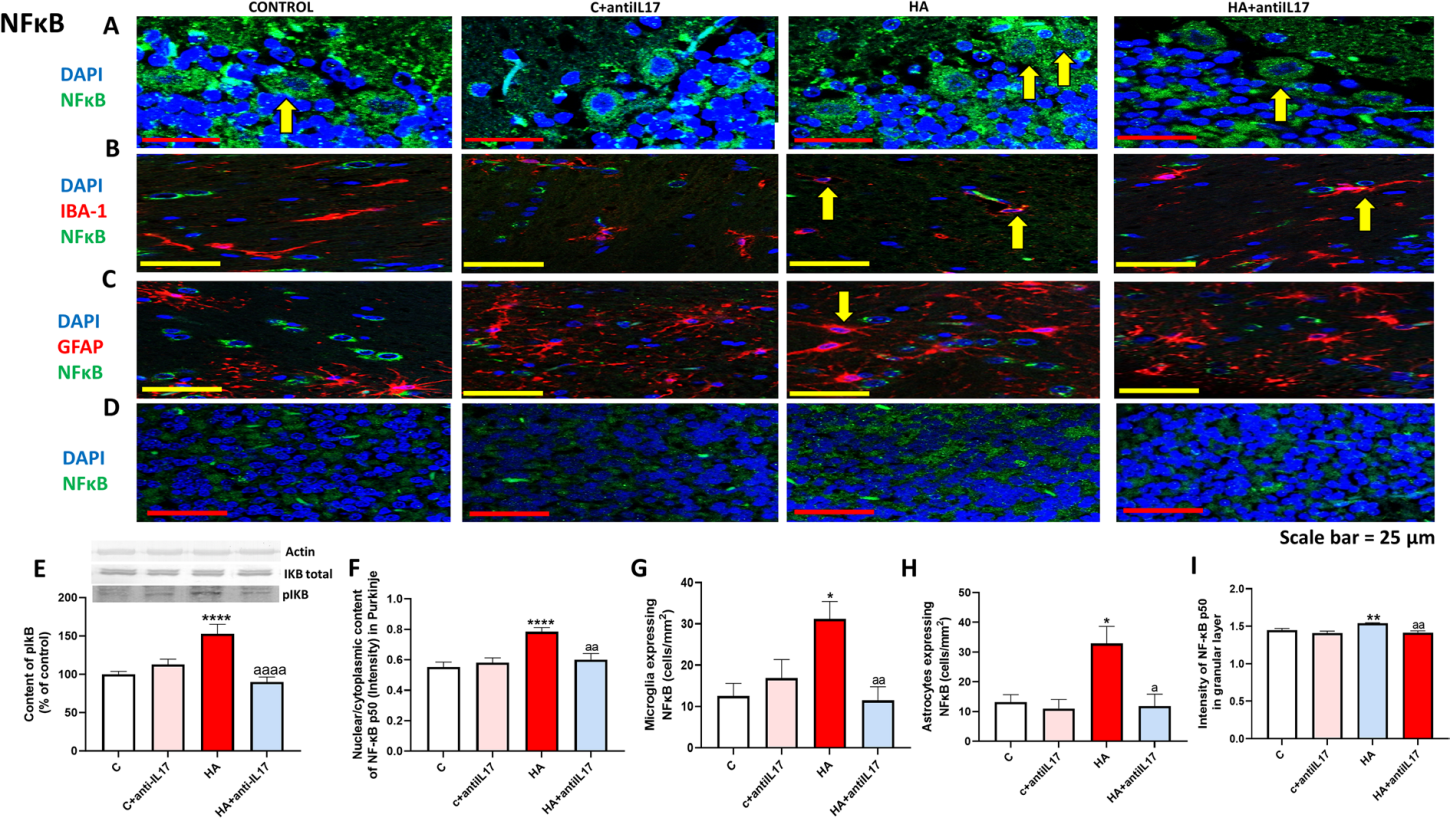
Ex vivo treatment with anti-IL-17 reverses the increase of NF-κB activation in Purkinje neurons, glia and granular neurons. Ex vivo treatment with anti-IL-17 reversed this activation. Nuclear translocation of NF-κB in Purkinje neurons (**A**, **F**) and the increase of NF-κB in microglia (**B**, **G**), astrocytes (**C**, **H**) and granular neurons (**D**, **I**) was analyzed by immunohistochemistry with DAB staining using antibodies against NF-κB (**A**, **D**) and double fluorescence staining using anti- IBA1 (**B**, **G**) or GFAP (**C**, **H**). Representative images of the immunohistochemistry and double fluorescence staining are shown (**A**–**D**). Protein content of IKBα (**E**) in cerebellar slices assessed by Western blot. Representative images of the blot are shown. Values are mean ± SEM of 4–6 rats per group in **A**–**D** and 20–24 in **E**. One-way ANOVA followed by Fisher’s LSD post-hoc test was performed to compare all groups. Values are the mean ± SEM. Values significantly different from control group are indicated by asterisk (*p < 0.05, **p < 0.01, ****p < 0.0001) and values significantly different from HA group are indicated by a (a = p < 0.05, aa = p < 0.01, aaaa = p < 0.0001). Yellow arrows mean colocalization of the two proteins while white arrows mean non-colocalization

Expression of TNFα is mainly modulated by the transcription factor NF-κB. We also analyzed the effects of hyperammonemia and of anti-IL-17 on NF-κB. Nuclear translocation of NF-κB was increased in Purkinje neurons of hyperammonemic rats and this is reversed by treatment with anti-IL-17 (Fig. 6A and F). Nuclear translocation of NF-κB is a consequence of the phosphorylation and release of IκB, which is increased in hyperammonemia and is reversed by anti-IL-17 (Fig. 6E). The amount of NF-κB is strongly increased in microglia (Fig. 6B and G) and astrocytes (Fig. 6C and H) of hyperammonemic rats and mildly in granular neurons (Fig. 6D and I). The increase of NF-κB was reversed by anti-IL-17 in all these cell types.

**Fig. 7:**
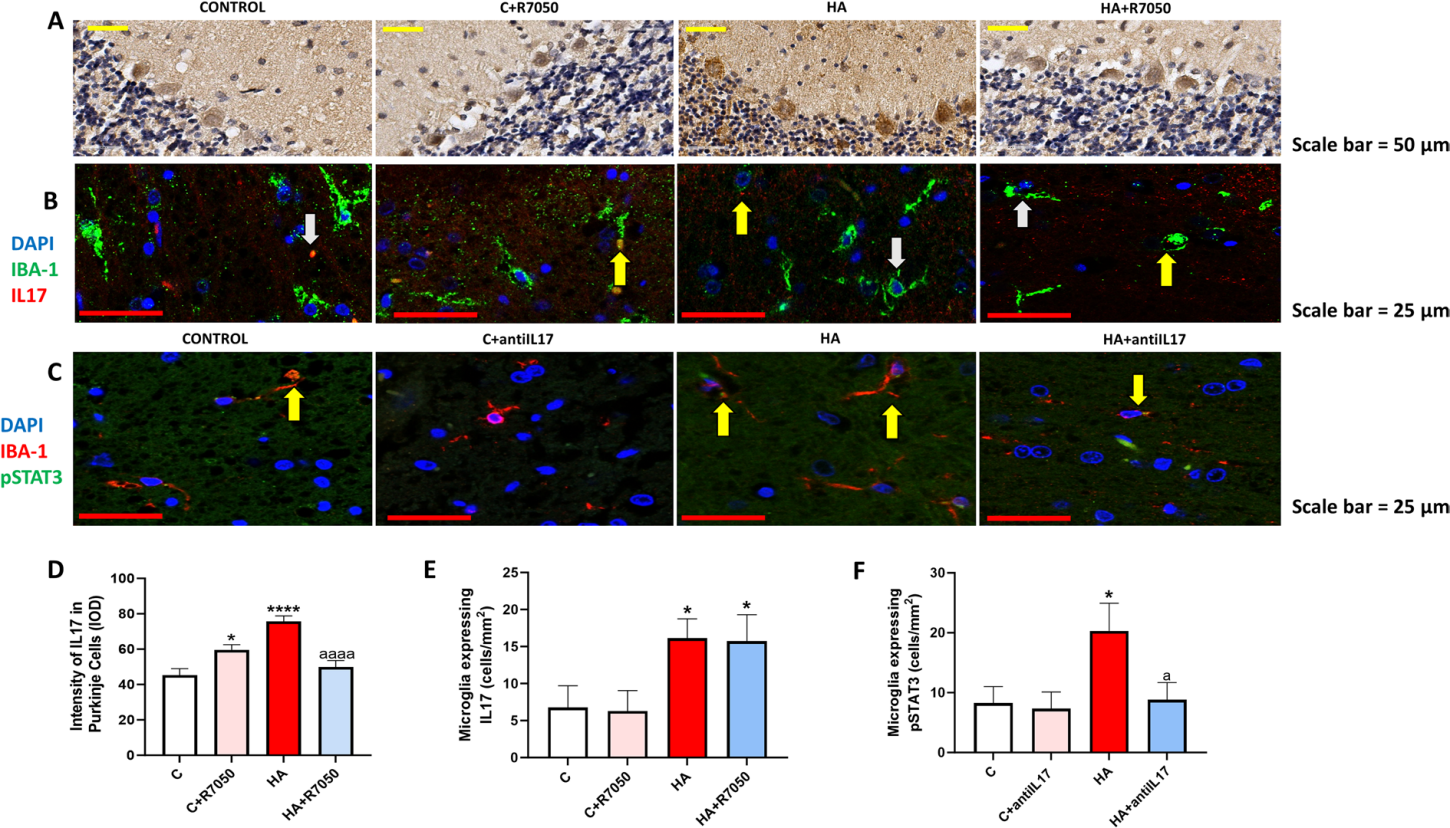
Blocking TNFR1 signaling ex vivo with R7050 reverses the increase of IL-17 in Purkinje neurons but not in microglia. Ex vivo treatment with anti-IL-17 reverses the increase of pSTAT3 in microglia in cerebellar slices from hyperammonemic rats. Ex vivo treatment with R7050 completely reversed the increase of IL-17 in Purkinje neurons (**A**, **D**) as analyzed by immunohistochemistry with DAB staining using antibodies against IL-17. Representative images of the immunohistochemistry staining are shown (**A**). The expression of IL-17 in microglia was analyzed by double immunofluorescence using antibodies against Iba1 and IL-17 (**B**, **E**). The expression of pSTAT3 in microglia was analyzed by double immunofluorescence using antibodies against Iba1 and pSTAT3 (**C**, **F**). Values are mean ± SEM of 4–6 rats per group. One-way ANOVA followed by Fisher’s LSD post-hoc test was performed to compare all groups. Values significantly different from controls are indicated by asterisk (*p < 0.05, ****p < 0.0001) and values significantly different from HA group are indicated by a (a < 0.05, aaaa = p < 0.0001). Yellow arrows mean colocalization of the two proteins

**Fig. 8:**
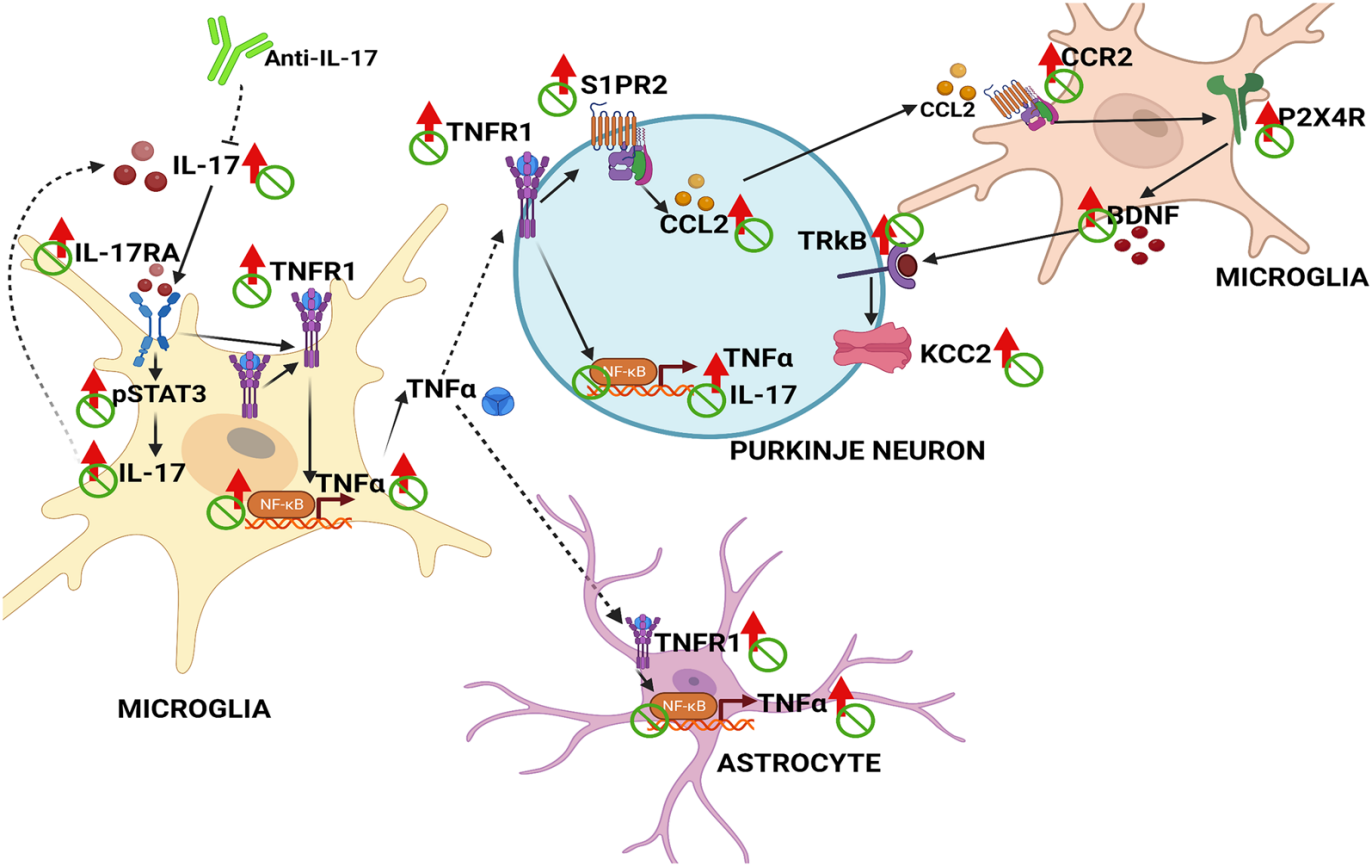
Proposed pathway by which increased IL-17 levels and activation of the IL-17 receptor in microglia induces neuroinflammation and activation of the TNFα-TNFR1-S1PR2-CCL2-BDNF-TrkB pathway in cerebellum of hyperammonemic rats. IL-17 levels and membrane expression and activation of the IL-17 receptor are enhanced mainly in microglia of hyperammonemic rats, leading to increased activation of STAT3 and NF-κB, which increase the formation of IL-17 and TNFα, respectively. TNFα released by microglia activates its receptor TNFR1 in Purkinje neurons, leading to activation of the TNFα-TNFR1-S1PR2-CCL2 pathway in Purkinje neurons. CCL2 released from Purkinje neurons activates CCR2 in microglia, inducing microglia activation and increased content of BDNF. BDNF released from microglia activates TrkB-mediated signals in neurons, leading to increased membrane expression of KCC2. This would enhance GABAergic neurotransmission and induce motor incoordination. The effects of hyperammonemia are indicated by red arrows (↑). Blockade with anti-IL-17 antibody is shown in green

We do not observe relevant expression of the IL-17 receptor in Purkinje neurons (Fig. 2B). This suggests that the reduction by anti-IL-17 of NF-κB activation and of TNFα and IL-17 levels in Purkinje neurons of hyperammonemic rats should be mediated by other factors. As the IL-17 receptor is expressed essentially in microglia, anti-IL-17 would prevent primarily the IL-17 receptor activation in microglia, thus reducing microglial IL-17 and TNFα in hyperammonemic rats. The TNFα released from microglia would activate TNFR1 in Purkinje neurons, leading to increased activation of NF-κB and content of TNFα and IL-17. To assess if this is the case we analyzed if blocking TNFR1 signaling with R7050 reverses the increase in IL-17 in Purkinje neurons. As shown in Fig. 7A and D, R7050 reversed the increase of IL-17 in Purkinje neurons of hyperammonemic rats. We have previously shown that R7050 also reverses the increased activation of NF-κB and content of TNFα in Purkinje neurons of hyperammonemic rats [25]. These data support that the TNFα released from microglia would activate TNFR1 in Purkinje neurons, leading to increased activation of NF-κB and content of TNFα and IL-17 (see Fig. 8).

As shown in Fig. 7B and E, blocking TNFR1 signaling with R7050 did not reverse the increase of IL-17 in microglia, indicating that it is not due to increased TNFα levels, but to increased IL-17 levels, as supported by the fact that it is reversed by anti-IL-17 (Fig. 1F–H). In contrast, R7050 completely reversed the increase of NF-κB and of TNFα in microglia [25]. The fact that R7050 does not reduce IL-17 in microglia, but reduces TNFα and NF-κB, suggests that the increase of IL-17 is not mediated by NF-κB but by some other transcription factor. It has been reported that STAT3 activation by phosphorylation increases IL-17 in microglia [17, 26]. We therefore analyzed the effects of hyperammonemia and of anti-IL-17 on phospho-STAT3 in microglia. As shown in Fig. 7C and F, increased activation of IL-17 receptor in hyperammonemia increases pSTAT3, which is completely reversed with anti-IL-17.

## Discussion

We have previously shown that enhanced GABAergic neurotransmission in cerebellum is responsible for motor incoordination in hyperammonemia and hepatic encephalopathy [6–8, 10, 27, 28] and that increased GABAergic neurotransmission is a consequence of neuroinflammation, especially of increased TNFα levels and increased activation of its receptor TNFR1 [10, 25].

We have previously shown that increased activation of TNFR1 leads to enhanced function of the TNFR1-S1PR2-CCL2-BDNF-TrkB pathway in cerebellum of hyperammonemic rats [10, 11]. Chronic hyperammonemia enhances TNFR1 and S1PR2 activation in the cerebellum by increasing its membrane expression. This increases CCL2, especially in Purkinje neurons. CCL2 activates CCR2 in microglia, leading to microglial activation, increased P2X4 membrane expression and BDNF in microglia. BDNF enhances TrkB activation in Purkinje neurons, leading to increased GAD65, GAD67 and GABA levels. Enhanced TrkB activation also increases the membrane expression of the γ2, α2 and β3 subunits of GABA_A_ receptors and of KCC2. Moreover, enhanced TrkB activation in activated astrocytes increases the membrane expression of GAT3 and NKCC1. All these effects are reversed in cerebellar slices from hyperammonemic rats ex vivo by blocking TNFR1 with R7050, S1PR2 with JTE-013, CCR2 with RS504393 or TrkB with ANA12, thus supporting a key role of the TNFR1-S1PR2-CCL2-CCR2-BDNF-TrkB pathway in enhancing GABAergic neurotransmission. Moreover, blocking S1PR2 in hyperammonemic rats by intracerebral administration of JTE-013 normalizes the S1PR2-CCL2-CCR2-BDNF-TrkB-KCC2 pathway, reduces glial activation and restores motor coordination in hyperammonemic rats [10, 11, 29–32].

However, the mechanism by which chronic hyperammonemia increases TNFα levels in cerebellum remained unknown. We show here that increased IL-17 levels and activation of the IL-17 receptor in microglia are responsible for the increased TNFα levels. Blocking the action of IL-17 with anti-IL-17 reversed the increase in TNFα levels in whole homogenates of cerebellum as well as in microglia, astrocytes and Purkinje neurons.

IL-17 levels are increased in microglia and in Purkinje and granular neurons but not in astrocytes of hyperammonemic rats. The IL-17 receptor is expressed in microglia, with negligible levels in neurons or astrocytes. Both the amount and the membrane expression of the IL-17 receptor are increased in hyperammonemia. This supports that hyperammonemia strongly enhances IL-17 signaling in microglia by increasing both the levels of IL-17 and the amount and membrane expression of the receptor. Enhanced activation of the IL-17 receptor increases the amount of the transcription factor NF-κB in microglia, leading to increased levels of TNFα which would mediate the effects on Purkinje neurons and astrocytes (Fig. 8). Enhanced activation of the IL-17 receptor in microglia also increases phosphorylation and activation of STAT3, which increases IL-17 transcription in microglia. IL-17 and TNFα are released from microglia to the extracellular fluid. However, IL-17 receptors are only present in microglia and not in Purkinje neurons or astrocytes. IL-17 would not mediate therefore the effects on these cells. These effects would be mediated by TNFα through the TNFR1 receptor, present in Purkinje neurons [25]. A similar activation of TNFα receptor in Purkinje neurons by TNFα produced in microglia has been reported by Kaur et al., [33].

Activation of TNFR1 induces activation of NF-kB and increased levels of IL-17 and TNFα in Purkinje neurons. This is supported by the fact that R7050, an antagonist of TNFR1 signaling [34] reverses the increase of TNFα [24, 25] and of IL-17 (as shown here) in Purkinje neurons. R7050 also reduces the increase of NF-kB and TNFα content in astrocytes of hyperammonemic rats [25], while IL-17 is not increased (Fig. 1E and I). Therefore, the sequence of events would be that summarized in Fig. 8. Hyperammonemia enhances the activation of the IL-17 receptor in microglia, leading to increased phosphorylation and activation of STAT3, which increases transcription of IL-17 in microglia, in agreement with previous reports [17, 26]. Activation of the IL-17 receptor in microglia also increases NF-kB, which increases TNFα. This TNFα generated in the microglia is released and increased extracellular TNFα activates TNFR1 in Purkinje neurons and astrocytes, leading to activation of NF-kB and increased levels of IL-17 and TNFα. In microglia extracellular TNFα increases activation of NF-kB and TNFα levels, but not IL-17.

Enhanced activation of TNFR1 in Purkinje neurons leads to activation of the TNFR1-S1PR2-CCL2-BDNF-TrkB pathway which mediates both microglia and astrocytes activation [10] and enhanced GABAergic neurotransmission [10, 11], which is responsible for motor incoordination [6–8].

The increase of IL-17 in cerebellum triggers therefore the process and signaling pathways leading to neuroinflammation, altered GABAergic neurotransmission and motor incoordination in rats with hyperammonemia and MHE. A key role of IL-17 in triggering neuroinflammation and pathological alterations has been already reported in different diseases [18].

Chen et al. [29] showed that, in a model of chronic migraine, peripheral IL-17 may cross the blood–brain barrier and reach the brain, where it activates microglia and induces neuroinflammation. In retinal vascular diseases there is an increase of IL-17 and of its receptor which result in enhanced activation of microglia which increases pro-inflammatory factors and contributes to the main pathological changes in retinal vascular diseases [17]. These authors propose that blockade of IL-17 could prevent vascular damages.

IL-17 also increases microglia activation and pro-inflammatory factors (IL-6, CCL2, BDNF) in primary cultures of microglia [15, 35] and following intraventricular IL-17A administration in mouse embryos [36].

Increased IL-17 levels contribute to the pathogenesis of several autoimmune and neurodegenerative diseases mediated by neuroinflammation. For example, in multiple sclerosis IL-17 act in the induction of oligodendrocyte cell death, neuronal dysfunction or axonal degeneration [37]. IL-17 seems to be also involved in the appearance of early cognitive deficits in Alzheimer's disease [38].

Liu et al. [39] showed that in rodent models of Parkinson’s diseases, the blood–brain barrier is disrupted and IL-17 levels increase in peripheral blood and substantia nigra. IL-17 activated microglia, increasing TNFα and IL-1β levels that facilitated dopaminergic neuron loss. Microglia expressed IL-17 receptor and this expression was upregulated by IL-17 (as we also observe here). IL-17 deficiency or intracerebral injection of anti-IL-17 alleviated blood–brain barrier disruption, microglial activation, neuronal loss and motor impairment.

Enhanced levels of IL-17 and of activation of its receptor also induce microglia activation and neuroinflammation, with increased TNFα and IL-1β brain levels, in a mice model of sepsis-associated encephalopathy. Neutralizing anti-IL-17 or anti-IL-17 receptor antibodies mitigated microglia activation and neuroinflammation and alleviated cognitive dysfunction [40].

IL-17 is also increased in vitreous of patients with retinal diseases and the IL-17 receptor is increased in microglia of rodent models of retinal vascular diseases. In retinal vascular diseases there is an activation of microglia by IL-17 which promotes endothelial cell growth, vascular leakage, and angiogenesis, the main pathological changes in retinal vascular diseases [17]. These authors propose that blockade of IL-17 could prevent vascular damages.

## Conclusions

These reports support a key role of increased IL-17 levels and of activation of its receptor in microglia in triggering neuroinflammation and neurological impairment in many different pathologies. We have previously shown that appearance of minimal hepatic encephalopathy (MHE), with mild cognitive and motor impairment, in patients with liver cirrhosis is associated with a shift in peripheral inflammation with increased activation of Th17 CD4 lymphocytes and increased plasma levels of IL-17 [12]. We show here that IL-17 levels are also increased in cerebellum of rats with hyperammonemia and MHE, leading to increased activation of IL-17 receptor in microglia, which triggers activation of STAT3 and NF-kB, leading to increased levels of IL-17 and TNFα, respectively. TNFα released from microglia then activates its receptor TNFR1 in Purkinje neurons, leading to activation of NF-kB and increased IL-17 and TNFα also in these cells. Enhanced activation of TNFR1 in Purkinje neurons also leads to activation of the TNFR1-S1PR2-CCL2-BDNF-TrkB pathway (Fig. 8) which mediates both microglia and astrocytes activation [10] and enhanced GABAergic neurotransmission [11], which is responsible for motor incoordination [10, 11]. IL-17 and IL-17 receptor in microglia would be therefore therapeutic targets to treat neurological impairment in patients with liver cirrhosis with MHE. We have previously shown that acting on other therapeutic targets within the TNFR1-S1PR2-CCL2-BDNF-TrkB pathway may also improve motor coordination in hyperammonemic rats [10, 11]. Exploring additional targets or combinatory approaches could enhance the efficacy of therapeutic interventions for MHE.

## Data Availability

All raw data used and analyzed for the current study are available from the corresponding author on reasonable request.

## Abbreviations

HE: Hepatic encephalopathy
MHE: Minimal hepatic encephalopathy
MI: Mean intensity
IOD: Integrated optical density (IOD)
